# 975 Implementation of RN Led RL Review Committee

**DOI:** 10.1093/jbcr/iraf019.506

**Published:** 2025-04-01

**Authors:** Nikole Williams, Rachael Underwood

**Affiliations:** University of Utah Health Burn Center; University of Utah Health

## Abstract

**Introduction:**

Our 2023 ABA Verification identified the need to track ongoing quality improvement projects and workflows for efficacy. Establishing a process for this specific need inspired the creation of this committee. RL (report and learn) Reports and Debriefing tools are sent to this committee for loop closure and tracking of quality improvement processes. This is a delegating committee that does not implement projects but filters them to the appropriate established workgroups throughout our burn center.

**Methods:**

The committee is comprised of RN lead, Shared governance committee chair, unit nurse management, APC representatives, and IDT members as needed. Monthly meeting with selected RL’s from unit CNCs containing recurrent themes appropriate to address on a unit level. The committee then decides the best course of action and which quality improvement project this data needs to be reported to for tracking of improvement. Each report is documented and scored using the trauma PIPS scoring scale and referred/escalated to the interdisciplinary QM committee as appropriate for system review.

**Results:**

Data from RL review has been categorized into common themes to identify gaps and track improvement for established workflows. This led to the hospital system building burn-specific automated dashboards for quality tracking with common themes. Management is now able to categorize RL’s into themes that line up with burn center process improvement practices as they are received. This allows us to track and pull data on an ongoing basis and identify gaps sooner, leading to better patient outcomes. The practice of reviewing debriefing tools allows burn management and senior staff to follow up with staff members that may need support after a stressful situation and help facilitate meaningful conversations within our team.

**Conclusions:**

This method used is sustainable, effective, and easily replicated.

**Applicability of Research to Practice:**

The goal of this workflow is to apply research to practice in a proactive approach by identifying trends early and updating established workflows to align with best practice. An example of this is applying the practice of a slower PACU recovery of pediatric patients. This committee responded to the need for nursing comfortability with pediatric airway management in a PACU setting by delegating this education need to our Skin Bud team who created a pediatric airway station. The following month we had an increase in pediatric PACU patients with lower Riker scores, and no RLs related to concerns with comfortability in maintaining open airways during recovery. This process allowed us to implement best PACU practice at the bedside in a safe way.

**Funding for the Study:**

N/A

